# Association of diabetes and perineural invasion in pancreatic cancer

**DOI:** 10.1002/cam4.43

**Published:** 2012-10-30

**Authors:** Ibrahim Halil Sahin, Mohamed A Shama, Motofumi Tanaka, James L Abbruzzese, Steven A Curley, Manal Hassan, Donghui Li

**Affiliations:** 1 Department of Gastrointestinal Medical Oncology, The University of Texas MD Anderson Cancer CenterHouston, Texas; 2 Department of Surgical Oncology, The University of Texas MD Anderson Cancer CenterHouston, Texas

**Keywords:** Diabetes, neuropathy, pancreatic cancer, perineural invasion

## Abstract

Diabetes and perineural invasion are frequently observed in pancreatic cancer. In this study, we tested possible relations between diabetes and perineural invasion in patients with resected pancreatic cancer. We conducted a retrospective study in 544 cases of resected pancreatic adenocarcinoma seen at the University of Texas MD Anderson Cancer Center during 1996–2011. Information on tumor characteristics, diabetes history, and survival time was collected by personal interview and medical record review. Patients with diabetes before or at the time of the pancreatic cancer diagnosis were considered diabetes only. Pearson χ^2^ test was used to compare categorical variables in diabetic and nondiabetic groups. Kaplan–Meier plot, log-rank test, and Cox proportional regression models were applied in survival analysis. The prevalence of diabetes and perineural invasion was 26.5% and 86.9%, respectively, in this study population. Patients with diabetes had a significantly higher prevalence of perineural invasion (92.4%) than those without diabetes (85%) (*P* = 0.025, χ^2^ test). Diabetes was not associated with other pathological characteristics of the tumor, such as tumor size, lymphovascular invasion, tumor grade, lymph node metastasis, and resection margin status. Diabetic patients had a significantly lower frequency of abdominal pain (*P* = 0.01), but a slightly higher frequency of weight loss (*P* = 0.078) as early symptoms of their cancer. Both diabetes and perineural invasion were related to worse survival and increased risk of death after adjusting for tumor grade and margin and node status (*P* = 0.036 and 0.019, respectively). The observed associations of diabetes and perineural invasion as well as reduced frequency of pain as early symptom of pancreatic cancer support the hypothesis that diabetes may contribute to pancreatic progression via the mechanism of nerve damage.

## Introduction

Pancreatic cancer is one of the most malicious human malignancies with the lowest 5-year survival rate [1]. Perineural invasion is frequently reported in pancreatic adeno-carcinoma [2] and is associated with aggressive tumor behavior and worse clinical outcome. Perineural invasion is defined as presence of cancer cells within the epineural, perineural, and endoneurial spaces of the neuronal sheet and around the nerves [3, 4]. This infiltration results in severe pain and nerve damage [5]. The reason why pancreatic cancer has a high frequency of perineural invasion is unknown.

Diabetes or impaired glucose tolerance is often concurrently present in patients with pancreatic cancer and is associated with worse prognosis [6]. Interestingly, nerve injury is a very well-known complication of diabetes which is characterized with neuroinflammation [7]. It has been hypothesized that hyperglycemia could promote perineural invasion in pancreatic cancer through two mechanisms: (1) enhanced cell proliferation and increased expression of cytokines such as nerve growth factors (NGFs) and enhanced interactions of nerve and cancer cells; (2) demyelinization and axonal degeneration of nerves, which facilitate cancer cells' invasion to the nerves [8]. A recent study of 61 resected pancreatic tumors has provided experimental evidence that hyperglycemia is associated with a higher expression of NGF in pancreatic cancer cells and p75 neurotrophin receptor (p75NTR) in nerve fibers, and these proteins may be involved in signaling between neurons and cancer cells which aggravate the process of perineural invasion [9].

To further examine the relationship of diabetes and perineural invasion, we conducted a large retrospective study in 544 surgically resected pancreatic ductal adenocarcinoma patients seen at University of Texas MD Anderson Cancer Center (MD Anderson) in the past 15 years. We observed significant associations of diabetes with a higher frequency of perineural invasion and a lower frequency of abdominal pain as early symptom of pancreatic cancer.

## Materials and Methods

### Study population

This retrospective study was conducted in 544 patients with resected pancreatic adenocarcinoma seen at MD Anderson from 1996 to 2011. Of the 544 cases, 374 were enrolled in a case–control study of pancreatic cancer conducted during 2000–2011 [10–12] and 170 were identified from the MD Anderson tumor registry. All cases had pathologically confirmed pancreatic adenocarcinoma. A total of 307 cases had their tumor resected at MD Anderson and 237 had tumor resection at different hospitals before seen at MD Anderson. The study was approved by the Institutional Review Board of MD Anderson.

### Data collection

Clinical and demographic information was collected from the medical records by trained personnel using a structured medical record abstraction form. Information on history of diabetes and other risk factors was also collected by personal interview for the 374 cases that were enrolled in the case–control study. Diabetes was defined as individuals with self-reported diabetes or use of antidiabetic medications at the time of recruitment to the case–control study or during the first clinical evaluation at MD Anderson. Patients who developed diabetes secondary to pancreatectomy or during the disease progression were not considered diabetic in this study. The presence or lack of perineural and lymphovascular invasion was determined by pathological evaluation of the resected tumor. Other pathological characteristics of the tumor, such as differentiation, resection margin status, lymph node metastasis, TNM stage and information on tumor site, tumor size, serum level of CA19-9 at cancer diagnosis, major early symptoms, date of cancer diagnosis, date of tumor resection, date of recurrence, date of last follow-up, or date of death, were also collected. The accuracy of the data abstraction was verified with repeated reading of randomly selected cases by different individuals. Dates of death were verified using at least one of the following sources: inpatient medical records, the MD Anderson tumor registry, and the Social Security Death Index.

### Statistical analysis

The distribution of demographic, clinical, and pathological characteristics was compared between diabetic and nondiabetic patients using the Pearson χ^2^ test. Overall survival (OS) time was calculated from the date of diagnosis to date of death. At the time of analysis, all living patients were censored. The association of OS with clinical variables was analyzed by Kaplan–Meier plot and log-rank test, and Cox proportional hazard regression models. Hazard ratios (HRs) and their 95% confidence intervals (CIs) were estimated in univariable model first, and variables with *P* < 0.05 were further evaluated in multivariable models. For all analyses, *P* < 0.05 was considered statistically significant. All statistical analysis was conducted using SPSS software, version 19 (SPSS, Chicago, IL).

## Results

The demographic and clinical characteristics of the study population are summarized in Table 1. A total of 144/544 (26.5%) patients reported a history of diabetes and none of these cases was known as type I diabetes. Patients with diabetes were overrepresented by older, male, minority, and overweight individuals. Perineural invasion was present in 86.9% of the cases. Diabetic patients (92.4%) had a significantly higher frequency of perineural invasion than nondiabetic patients (85%) (*P* = 0.025). When the analysis was restricted to whites only, the result remained almost the same (*P* = 0.016). No significant difference was observed for lymphovascular invasion, resection margin, and node status, as well as tumor size and grade between diabetic and nondiabetic patients (Table 1). As reported in previous studies [13, 14], a greater proportion of diabetic patients (46.5%) received neoadjuvant therapy than nondiabetic patients (41.8%); diabetic patients had a higher frequency of tumors located at the pancreas body or tail than nondiabetic patients did. Notably, diabetic patients had a significantly lower frequency of reporting abdominal pain as an early symptom of the cancer than nondiabetic patients did (Table 2). Diabetic patients also had a slightly higher frequency of weight loss than nondiabetics (Table 2); diabetics lost an average 7.5 pounds more than nondiabetics did among those who reported weight loss. Furthermore, perineural invasion was associated with a significantly higher frequency of distant metastasis or local recurrence while such associations were not observed for diabetes (Table 3).

**Table 1:** Patient characteristics by diabetes status

Tumor characteristics	All patients, *N* (%)	Nondiabetic, *N* (%)	Diabetic, *N* (%)	*P*
Age (years)				
<50	60 (11.0)	52 (13.0)	8 (2.1)	0.075
50–60	161 (29.6)	120 (30.0)	41 (31.9)	
60–70	207 (38.1)	147 (36.8)	60 (66.0)	
>70	116 (21.3)	81 (20.3)	35 (24.3)	
Race				
White	473 (86.9)	363 (90.8)	110 (76.4)	<0.001
Black	22 (4.0)	12 (3.0)	10 (6.9)	
Hispanic	33 (6.1)	15 (3.8)	18 (12.5)	
Others	16 (2.9)	10 (2.5)	6 (4.2)	
Sex				
Male	314 (57.7)	221 (55.3)	93 (64.6)	0.052
Female	230 (42.3)	179 (44.8)	51 (35.4)	
BMI				
<25	228 (44.4)	178 (47.2)	50 (36.8)	0.005
25.1–30	197 (38.4)	146 (38.7)	51 (37.5)	
>30	88 (17.2)	53 (14.1)	35 (25.7)	
Perineural invasion				
No	71 (13.1)	60 (15.0)	11 (7.6)	0.025
Yes	473 (86.9)	340 (85.0)	133 (92.4)	
Lymphovascular invasion				
No	177 (35.3)	137 (36.9)	40 (30.5)	0.188
Yes	325 (64.7)	234 (63.1)	91 (69.5)	
T stage				
T1	43 (7.9)	35 (8.8)	8 (5.6)	0.248
T2	23 (4.2)	20 (5.0)	3 (2.1)	
T3	468 (86.2)	337 (84.5)	131 (91.0)	
T4	9 (1.7)	7 (1.8)	2 (1.4)	
Node status				
No	190 (34.9)	135 (33.8)	55 (38.2)	0.337
Yes	354 (65.1)	265 (66.3)	89 (61.8)	
Margin status				
Negative	426 (78.3)	315 (78.8)	111 (77.1)	0.677
Positive	118 (21.7)	85 (21.3)	33 (22.9)	
Differentiation				
Well–moderate	302 (56.9)	227 (58.1)	75 (53.6)	0.358
Poor	229 (43.1)	164 (41.9)	65 (46.4)	
Tumor site				
Pancreas head and neck	439 (82.2)	327 (83.2)	112 (79.4)	0.315
Body and tail	95 (17.8)	66 (16.8)	29 (20.6)	
Tumor size (cm)				
<2	145 (27.8)	109 (28.4)	36 (26.1)	0.620
2.1–3.0	192 (36.2)	137 (35.7)	52 (37.7)	
>3.0	186 (36.0)	136 (36.0)	50 (36.2)	
CA19-9 (U/mL)				
<47	152 (33.8)	119 (36.0)	33 (27.7)	0.160
48–1000	249 (55.3)	180 (54.4)	69 (58.0)	
>1000	49 (10.9)	32 (9.7)	17 (14.3)	

Numbers do not add to the total number of patients for BMI, lymphovascular invasion, T stage, tumor grade, tumor site, and CA19-9 because of missing information.

**Table 2:** Clinical symptoms at diagnosis by diabetes status

Symptoms	All patients, *N* (%)	Nondiabetic, *N* (%)	Diabetic, *N* (%)	*P*
Abdominal pain				
No	260 (47.8)	178 (44.5)	82 (56.9)	0.010
Yes	284 (52.2)	222 (55.5)	62 (43.1)	
Jaundice				
No	238 (43.8)	176 (44.0)	62 (43.1)	0.845
Yes	306 (56.3)	224 (56.0)	82 (56.9)	
Anorexia				
No	491 (90.3)	361 (90.3)	130 (90.3)	0.992
Yes	53 (90.7)	39 (9.8)	14 (9.7)	
Weight loss				
No	81 (19.3)	65 (21.8)	16 (13.8)	0.078
Yes	339 (80.7)	239 (78.6)	100 (69.4)	

Information on weight loss is missing in 124 patients.

**Table 3:** Tumor recurrence by diabetes and perineural invasion status

	Diabetes, *n* (%)		Perineural invasion, *n* (%)	
		
Recurrence status	No	Yes	No	Yes
Not recurred	114 (29.0)	43 (30.3)	34 (49.3)	123 (26.5)
Local	68 (17.3)	18 (12.7)	9 (12.7)	77 (16.6)
Metastatic	211 (53.7)	81 (57.0)	27 (38.0)	265 (57.0)
*P* (χ^2^ test)	0.436			0.001				

The median OS time for the entire study population was 31.3 months (95% CI, 28.6–34.0). Diabetes, presence of perineural invasion, positive margin and node status, and poor differentiation were significantly associated with reduced survival and increased risk of death in this patient population (Table 4). Baseline serum level of CA19-9 and presence of lymphovascular invasion were also significant predictors for reduced survival, but they were not included in the multivariable models because of missing values in a large number of patients. In the final multivariable Cox regression model, diabetes was associated with 29% increased risk for death (*P* = 0.036) and perineural invasion had a HR of 1.60 and 95% CI of 1.08–2.36 (*P* = 0.019).

**Table 4:** Overall survival and risk of death by clinical characteristics

			Univariate		Multivariate^1^	
				
Condition	Median survival time (months)	*P* value (log-rank)	HR (95% CI)	*P*	HR (95% CI)	*P*
Diabetes						
No	33.2 ± 1.8	0.049	1.0	0.050	1.0	0.036
Yes	24.6 ± 2.5		1.27 (1.00–1.60)		1.29 (1.02–1.64)	
Perineural invasion						
No	51.7 ± 31.1	<0.001	1.0	<0.001	1.0	0.019
Yes	29.4 ± 1.64		2.01 (1.38–2.93)		1.60 (1.08–2.36)	
Margin status						
Negative	33.6 ± 1.7	<0.001	1.0	<0.001	1.0	0.003
Positive	24.8 ± 2.6		1.62 (1.27–2.08)		1.47 (1.14–1.90)	
Node status						
Negative	38.6 ± 5.3	<0.001	1.0	<0.001	1.0	0.002
Positive	27.5 ± 1.3		1.70 (1.35–2.15)		1.45 (1.14–1.83)	
Differentiation						
Well–moderate	35.9 ± 2.68	<0.001	1.0	<0.001	1.0	<0.001
Poor	26.0 ± 1.77		1.63 (1.32–2.02)		1.63 (1.32–2.02)	

1 All variables in the table were included in the multivariate model.

## Discussion

In this retrospective study, we have observed that diabetic patients with resected pancreatic adenocarcinoma had a significantly higher prevalence of perineural invasion and lower frequency of abdominal pain as the early symptom of pancreatic cancer. These observations support the hypothesis that diabetes may contribute to pancreatic cancer and aggravate the process of perineural invasion via the mechanisms of nerve damages and inflammation.

**Figure 1:**
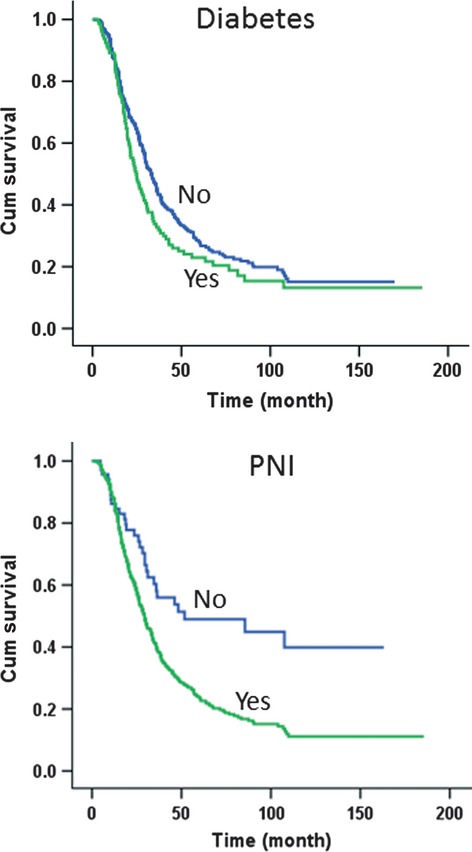
Overall survival curve by diabetes (upper panel) and perineural invasion (PNI) status (lower panel). *P* value was 0.049 for diabetes and <0.0001 for perineural invasion (log-rank test).

Perineural invasion is defined as the presence of cancer cells along nerves and/or within the epineurial, perineurial, and endoneurial spaces of the neuronal sheath [4]. Perineural invasion is a multifactorial process that involves various signaling molecules from different signaling pathways [3, 15, 16]. These signaling molecules include NGFs, neurotrophic factors, proteinases, cytokines, chemokines, and cell-surface ligands and receptors [17–19]. Pancreatic ductal adenocarcinomas cells have a strong neurotrophic effects, and the pancreas is in close proximity to several neural plexuses, which may partially explain the high incidence of perineural invasion in pancreatic cancer [2, 20]. On the other hand, neuropathy is a well-known complication of diabetes and is associated with injury to myelin sheet and neuroinflammation [7]. Neurons have a less rapidly glucose uptake than endothelial cells, which may account for the high susceptibility of neurons to glucose-mediated injury [21]. It is conceivable that under hyperglycemic conditions, increased level of oxidative stress and proinflammatory factors cause nerve damages and inflammatory responses [22], which simultaneously facilitate cancer cell proliferation, migration, and metastasis [23]. The clinical association observed in the current study is supported by the previously reported experimental evidence that hyperglycemia was related to a higher expression of NGF and neurotrophic factors in pancreatic cancer cells and nerve fibers [9]. Thus, the high prevalence of diabetes and impaired glucose tolerance in pancreatic cancer may also play a role in the development of perineural invasion (Fig. 1).

In many cases, perineural invasion is accompanied by pain, and many of the molecules involved in perineural invasion are also implicated in pain generation [5, 24]. However, we observed a significantly lower frequency of abdominal pain in diabetic patients as early symptom of pancreatic cancer in this study. It is known that diabetic neuropathy could be peripheral, autonomic, and proximal or focal. Peripheral neuropathy may result in both numbness and painful symptoms, and autonomic neuropathy may lead to various symptoms including silent (painless) myocardial infarction [25]. While loss of sensation may increase risk of feet damage, whether decreased frequency of initial symptom of abdominal pain in diabetic patients may delay the diagnosis of pancreatic cancer needs further investigation.

The strengths of our study are large sample size of resected pancreatic ductal adenocarcinoma patients and available detailed pathological and clinical information. The main limitations, however, were related to the retrospective design and associated recall and information bias. Because many patients may have pancreatic cancer-caused diabetes but were not aware of it and we did not perform clinical test to confirm the diabetes diagnosis, there could be misclassification bias. We also failed to consider diabetes developed after the cancer diagnosis or during the cancer treatment and its impact on perineural invasion and pancreatic pain. Future mechanistic studies or experimental studies are required to fully elucidate the relations between diabetes and perineural invasion in pancreatic cancer.
